# Non-islet cell tumor-induced hypoglycemia: a report of five cases and brief review of the literature

**DOI:** 10.1530/EDM-13-0046

**Published:** 2013-11-20

**Authors:** Pinaki Dutta, Anuradha Aggarwal, Yashpal Gogate, Uma Nahar, Viral N Shah, Mandeep Singla, N Khandelwal, Anil Bhansali

**Affiliations:** 1 Department of EndocrinologyPost Graduate Institute of Medical Education and ResearchChandigarh, 160012India; 2 Department of HistopathologyPost Graduate Institute of Medical Education and ResearchChandigarh, 160012India; 3 Department of RadiodiagnosisPost Graduate Institute of Medical Education and ResearchChandigarh, 160012India

## Abstract

We describe the clinical presentation, diagnostic and management issues in five cases of non-islet cell tumor hypoglycemia (NICTH), diagnosed at a tertiary care institute over a period of 15 years. The clinical, laboratory, and histopathological findings of these patients along with diagnostic utility of IGF2:IGF1 ratio are discussed. The mean age of presentation was 52 years, with a male predominance (3:2). Three patients presented with recurrent episodes of fasting hypoglycemia and it was detected in other two patients during hospitalization. Two patients had acromegaloid features that regressed following treatment. One patient had hypokalemia. Low levels of insulin, C-peptide, GH, and IGF1 were invariably found in all. The IGF2 level was elevated in only one patient; however, IGF2:IGF1 ratio was more than 10 in four of the five patients. The mean tumor size was 16.4 cm and mean weight was 3.6 kg. Four patients had mesenchymal tumors and one had epithelial tumor. NICTH is a rare cause of hypoglycemia. Hypoinsulinemic hypoglycemia with low IGF1 and IGF2:IGF1 ratio more than 10 is suggestive of this entity.

**Learning points:**

- NICTH should be considered in patients presenting with tumor of mesenchymal origin and hypoglycemia.
- Hypoinsulinemic hypoglycemia with low IGF1 is a strong biochemical evidence of NICTH.
- IGF2:IGF1 ratio of more than 10 is a complementary investigation in the absence of an assay facility for IGF2.

## Background

Hypoglycemia in a non-diabetic is defined as a plasma glucose level <45 mg/dl and is to be considered significant only when it is accompanied with symptoms suggestive of hypoglycemia. Hypoglycemia is a medical emergency and is mostly iatrogenic in patients with diabetes. Tumor-related hypoglycemia can be induced by excessive secretion of insulin by islet cell tumors (insulinoma and neuroendocrine tumors), insulin-like growth factor 2 (IGF2) from mesenchymal and epithelial tumors, and rarely by secretion of IGF1, cytokines, catecholamines or increased tumor metabolism of glucose *per se*. Non-islet cell tumor hypoglycemia (NICTH) is a very rare cause of hypoglycemia. It mainly occurs in patients with solid tumors of mesenchymal and epithelial origin, which secrete partially processed IGF2 (big IGF2) (1). We describe five such cases seen in a tertiary care center over the last 15 years with brief review of the literature.

## Case presentation

### Case 1

**Figure 1:**
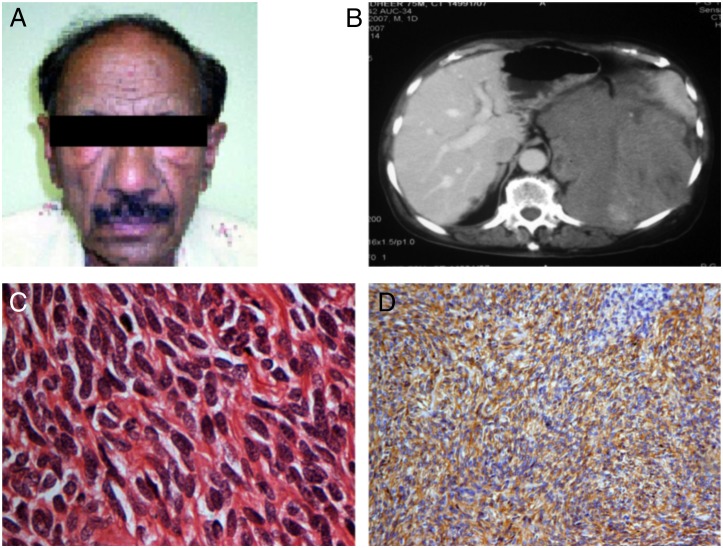
(A) Clinical photograph of patient showing acromegaloid features and multiple skin tags. (B) CECT of the abdomen showing large heterogenous mass. (C) Low-power photomicrograph (10×, H&E) showing hypercellular lesions arranged in storiform pattern. Cells with scanty cytoplasm, hyperchromatic nuclei, and occasional mitotic figures are seen. (D) Immunostaining showing positivity for CD34. Reproduced with permission from the BMJ Publishing Group Ltd. Dutta P, Bhansali A, Santosh Kumar P, Jayaprakash P & Nahar U (2009) A rare cause of hypoglycemia. *BMJ Case Reports*. doi:10.1136/bcr.04.2009.1789

A 77-year-old male presented with weight loss, episodic altered sensorium, and mass in abdomen for 2 months. Computer tomographic (CT) scan of the abdomen revealed a 16×16 cm retroperitoneal mass extending to the kidney and lateral chest wall (Fig. 1). During hospitalization, he lost consciousness with blood glucose of 28 mg/dl. Ten years before, he had undergone thoracotomy and excision of a posterior mediastinal mass, which weighed 2 kg and was then reported as fibroma. During that presentation, he had no history or documented episode of hypoglycemia. On examination, he had multiple skin tags and acromegaloid features. On extended fasting, he developed hypoglycemic symptoms with blood glucose of 37 mg/dl at 3 h, plasma insulin 1.5 μU/ml (normal:<6 μU/ml), growth hormone (GH) 0.54 ng/ml, IGF1 48 ng/ml (normal: 94–190 ng/ml), and IGF2 1133 ng/ml (normal: 440–1249 ng/ml). Fine-needle aspiration cytology (FNAC) was suggestive of a fibroma. He was started on complex carbohydrate diet, prednisolone 20 mg/day with which his symptoms abated and was subjected to surgical debulking. Histopathology revealed some areas of atypia suggestive of sarcomatous change. In view of aggressiveness of the tumor and residual disease, he received local radiotherapy (20 Gy) in fractions and six cycles of combination chemotherapy (vincristine+cyclophosphamide+doxorubicin). Repeat CT scan revealed residual disease. On follow-up, he was on prednisolone 7.5 mg/day with no recurrence of symptoms for the next 6 years and later lost to follow-up.

### Case 2

**Figure 2:**
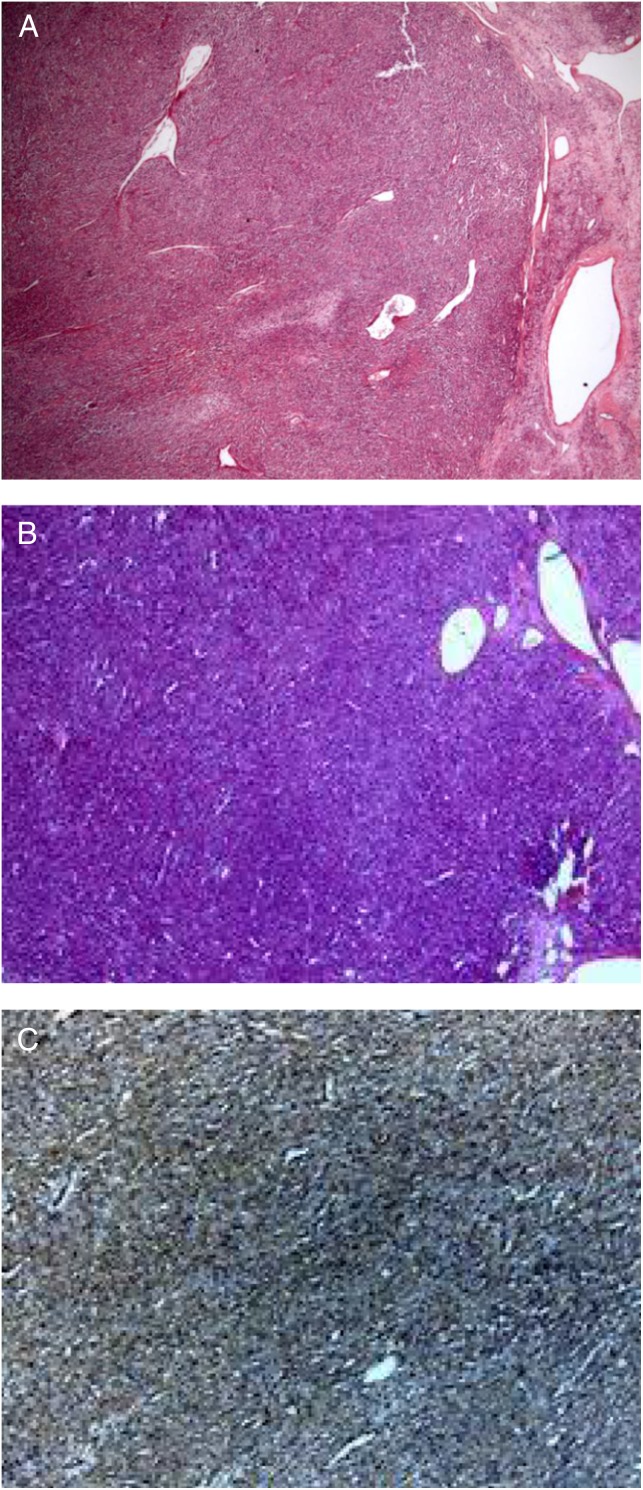
(A) Low-power photomicrograph (10×, H&E) showing numerous capillary sized vessels lined by flattened epithelial cells, surrounded by sheets and nests of oval, elongated tumor cells. Mitosis is 4–5/10 HPF. Note is made of large areas of hyalinization and focal areas of necrosis. (B) PA stain highlights vascular pattern of tumor. (C) Immunostaining showing positivity for CD34.

A 24-year-old female presented with 6-month history of hunger, sweating, and tremors, which used to subside immediately after food intake. She had documented blood glucose values below 40 mg/dl during those episodes. On evaluation, she had a palpable mass in the abdomen and a CT scan of the abdomen revealed a well-defined lobulated, heterogeneous, hypervascular mass in the left upper abdomen measuring 15×8×8 cm with the epicenter in the retroperitoneum. The mass was abutting left adrenal gland, spleen, and celiac axis. The FNAC of the mass was suggestive of sarcoma. She received three cycles of chemotherapy and was referred to our institute. At blood glucose of 25 mg/dl, she had undetectable plasma insulin (≤0.2 μU/ml), low C-peptide 0.084 ng/ml (normal: 1.1–4.4 ng/ml), normal cortisol level (615 nmol/l; normal:>550 nmol/l during stress), and a GH level of 0.2 ng/ml. She had low IGF1 <25 ng/ml (normal: 116–358 ng/ml); with IGF2 level of 541 ng/ml (normal: 288–736 ng/ml) and high IGF2:IGF1 ratio of 21.64 (normal:<10). She underwent laparotomy and *en bloc* resection of the retroperitoneal mass with splenectomy, distal pancreatectomy, and left nephrectomy. Histopathology was suggestive of hemangiopericytoma (Fig. 2). After surgery, there was no recurrence of hypoglycemia. She is doing well for the last 3 years and follow-up ^18^FDG–PET scan is normal.

### Case 3

**Figure 3:**
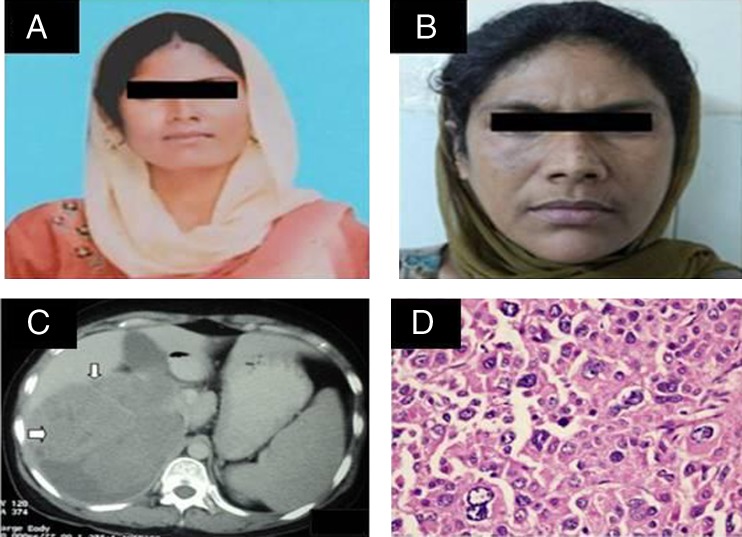
(A) Previous photograph of patient. (B) Photograph of patient showing thick lips, bumpy nose, and coarse facial features. (C) CECT of the patient showed large heterogenous right adrenal mass (15×12×12 cm). (D) Histopathology of right adrenal mass showing moderate degree of pleomorphism, hyperchromatic nucleus, stippled chromatin, prominent nucleoli, and mitosis (H&E, 40×).

A 32-year-old female presented with recurrent episodes of neuroglycopenic symptoms for 2 weeks especially during the fasting state. On examination, she had coarse facial features, acral enlargement, seborrhea, and acanthosis nigricans. At 9 h of extended fast, her blood glucose was 38 mg/dl with undetectable insulin (≤0.2 μU/ml) and low C-peptide level (0.08 ng/ml; normal: 1.1–4.4 ng/ml), normal cortisol level (561 nmol/l; normal:>550 nmol/l during stress), and a GH level of 4.2 ng/ml. She had low IGF1 56.9 ng/ml (normal: 115–307 ng/ml), with IGF2 level of 489 ng/ml (normal: 288–736 ng/ml) and normal IGF2:IGF1 ratio of 8.6 (normal:<10). Serum GH was suppressible (<1 ng/ml) after glucose load. Contrast enhanced CT scan of the abdomen revealed a large lobulated, heterogenous, hypervascular right suprarenal mass of size 15.3×12.7×12 cm with necrosis and inferior vena cava compression without any local invasion. Her cortisol dynamics and urinary metanephrines were normal. She underwent open right adrenalectomy, following which hypoglycemic episodes ameliorated and her acromegaloid facial features regressed. Histopathology revealed adrenocortical carcinoma (Fig. 3). Follow-up contrast enhanced CT (CECT) scan of the chest and abdomen revealed lung metastasis. Patient is not able to afford mitotane-based chemotherapy; however, she is asymptomatic and under regular follow-up.

### Case 4

**Figure 4:**
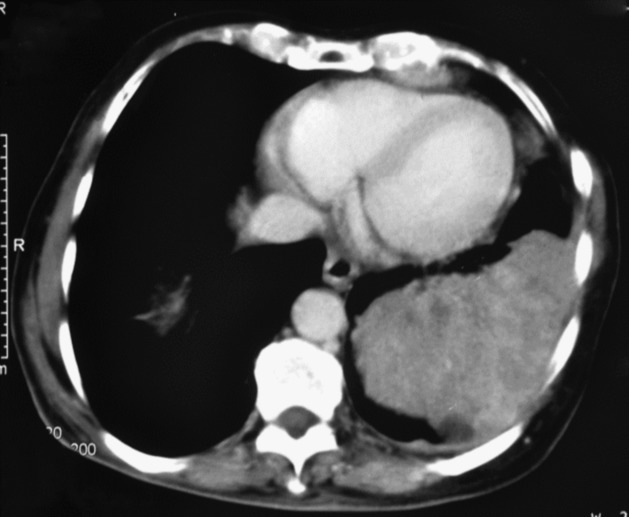
CECT scan of patient showing large heterogenous mass in left lower lung field with necrosis and calcification.

An 80-year-old male chronic smoker, hypertensive, and non-diabetic with no exposure to hypoglycemic agents presented with cough, breathlessness, recurrent episodes of neuroglycopenia, and seizures for 6 weeks. He had documented low blood glucose of 33 mg/dl during those episodes. He had digital clubbing with decreased air entry in left infrascapular region. During hospitalization, he developed neuroglycopenia with blood glucose of 33 mg/dl, plasma insulin <0.200 μU/ml, C-peptide 0.687 ng/ml (normal: 1.1–4.4 ng/ml), cortisol 446 nmol/l (normal:>550 nmol/l at the time of stress), and GH of 1.2 ng/ml. He had low IGF1 <25 ng/ml (normal: 55–166 ng/ml), with IGF2 level of 860 ng/ml (normal: 288–736 ng/ml) and IGF2:IGF1 ratio of 34.4 (normal:<10). CT of abdomen showed 21×20×12.5 cm mass in the left lower lung field with necrosis and calcification (Fig. 4). Ultrasound-guided FNAC was suggestive of pleural mesothelioma. Patient declined surgery and chemotherapy and was started on prednisolone 20 mg/day with which his hypoglycemic episodes subsided. However, he was lost to follow-up.

### Case 5

**Figure 5:**
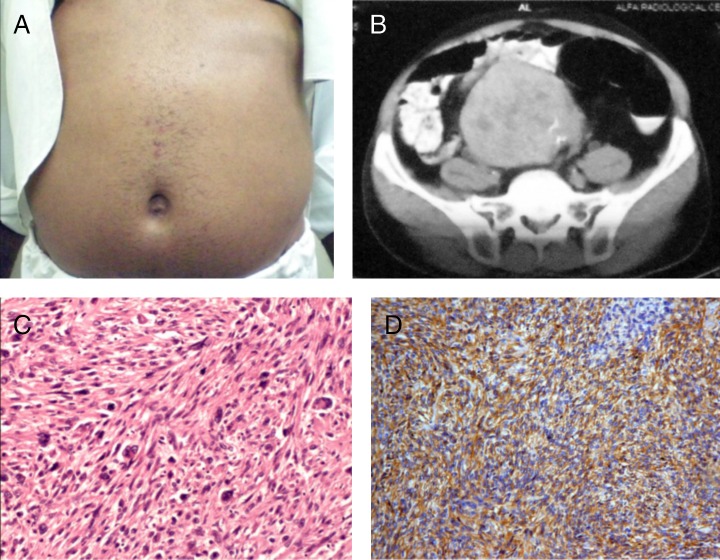
(A) Clinical photograph of patient. (B) CECT abdomen of patient showing inhomogeneously enhancing retroperitoneal mass arising from pelvis extending superiorly till umbilicus. (C) Low-power photomicrograph (10×, H&E) showing short fascicles of spindle cells with intervening collagen suggestive of solitary fibrous tumor. (D) Immunostaining showing positivity for CD34.

A 48-year-old male was admitted with history of recurrent abdominal pain one and a half years. Ultrasonography of the abdomen revealed a large retroperitoneal mass. He was admitted for surgery. On the day of surgery, he developed delirium following overnight fasting and surgery was canceled. Patient was started on i.v. glucose following which he improved. On the day of next surgery, patient developed similar features with right-sided hemiparesis. During this episode, blood glucose of 40 mg/dl was documented and endocrinology consultation was sought. On further evaluation, after 8 h of extended fast, he had blood glucose of 31 mg/dl with corresponding plasma insulin of <0.2 μU/ml (normal:<6 μU/ml), GH <0.05 ng/ml, IGF1 56 ng/ml (normal: 94–252 ng/ml), IGF2 623 ng/ml (normal: 288–736 ng/ml), and IGF2:IGF1 ratio of 11.1 (normal:<10). A diagnosis of hypoinsulinemic hypoglycemia was made. CECT scan of the abdomen revealed 15.2×9.2×9.5 cm homogenously enhancing retroperitoneal mass arising from the pelvis extending superiorly up to the umbilicus (Fig. 5). He underwent surgical removal of the tumor. Histopathology was suggestive of leiomyosarcoma. At 6 months, repeat imaging did not reveal any residual or recurrent disease. He is asymptomatic and under regular follow-up.

## Investigation

- Patient with NICTH will have hypoinsulinemic hypoglycemia and low GH and β-hydroxybutyrate.
- Total IGF2, as determined by conventional immunometric or receptor assays, may be either increased, decreased, or within the normal range.
- More than 60% of IGF2 is in a higher molecular weight, i.e. big IGF2, as determined by specific immunometric assays.
- Inappropriately reduced levels of IGF1 in a patient having hypoinsulinemic hypoglycemia strongly points toward the diagnosis of NICTH.
- Increased ratio between total IGF2 and IGF1 >10 is a useful additional marker where facility for measuring big IGF2 is not available.
- They are easily detected by conventional radiological imaging. Functional imaging like FDG–PET may lead to false negative result due to accelerated uptake of FDG by skeletal and cardiac muscles as these tissues have much higher expression of insulin receptor when compared with tumor tissue.

A summary of the clinical and biochemical profiles of all the cases presented in this reported is presented in Table 1.

**Table 1:** Clinical and biochemical profile of all the patients

	**Case 1**	**Case 2**	**Case 3**	**Case 4**	**Case 5**
Age (years)	77	24	32	80	48
Sex (M/F)	M	F	F	M	M
Lag period (weeks)	8	24	2	6	78
Glucose (mg/dl)	28	25	38	33	31
Insulin (μU/ml)	1.5	<0.2	<0.2	<0.2	<0.2
Insulin:glucose ratio (μU/ml per mg per dl)	0.05	0.008	0.005	0.006	0.006
GH (ng/ml)	0.54	0.2	4.2	1.2	0.5
IGF1 (ng/ml)	48	<25	56.9	<25	56
IGF2 (ng/ml)	1133	541	489	860	623
IGF2:IGF1	23.6	21.64	8.6	34.4	11.1
Tumor weight (kg)	4.8	2.5	3.8	–	3.4
Tumor size (cm)	16×16×16	15×8×8	15×12×12	21×20×12.5	15.2×9.2×9.5
Histopathology	Fibroma	Hemangiopericytoma	Adrenocortical carcinoma	Pleural mesothelioma	Leiomyosarcoma
Intervention	Surgery+chemotherapy+RT+prednisolone	Chemotherapy followed by surgery	Surgery	Oral prednisolone	Surgery

## Treatment

The long-term therapeutic strategies in NICTH involve complete removal of the tumor or reduction of the tumor mass. The metabolic alterations caused by NICTH are fully reversible after successful surgical removal of the ‘big’-IGF2-producing tumor. When curative resection is no longer possible, other approaches like chemotherapy directed against the tumor or selective embolization of tumor may be helpful. In one of our patients, follow-up CT scan showed residual disease and review of histopathology revealed some areas of sarcomatous changes. Glucocorticoids provide relief from hypoglycemia by stimulating glyconeogenesis and gluconeogenesis. It also suppresses the production of ‘big’-IGF2 at least in some cases. Moderate to high doses of glucocorticosteroids may cause shrinkage of the tumor. Administration of rhGH is considered to be beneficial in the treatment of NICTH. It ameliorates hypoglycemia by stimulating hepatic gluconeogenesis and glycogenolysis.

## Outcome and follow-up

Although this study was performed for the last 15 years, patients were seen at various points of time (Table 2).

**Table 2:** Follow-up of study patients at various time points

**S. no.**	**Year when first seen**	**Follow-up**
1	1998	Repeat CT scan after 1 year of chemotherapy revealed residual disease. On prednisolone 7.5 mg, he was asymptomatic for 6 years and later lost to follow-up
2	2002	She was asymptomatic during her follow-up of 3 years. FDG–PET scan, on follow-up was also normal
3	2010	Having regular follow-up, she is asymptomatic. Follow-up CECT scan of the chest and abdomen revealed lung metastasis. Patient cannot afford mitotane-based chemotherapy
4	2010	Lost to follow-up
5	2013	Having regular follow-up. He is asymptomatic. Repeat imaging at 6 months did not reveal any residual or recurrent disease

## Discussion

NICTH is a rare paraneoplastic syndrome and is the second most common cause of tumor-related hypoglycemia following insulinoma. It was first described in 1929 in a patient with hepatocellular carcinoma (2). Daughaday *et al*. (1988) (1) showed for the first time that tumor-induced hypoglycemia was associated with the aberrant production of pro-IGF2 (big-IGF2). Its prevalence is not known and is likely that many cases would go undiagnosed. In the current small series of five cases seen at a single center, we describe the diversity in clinical presentation, the therapeutic problem, and the role of IGF2:IGF1 ratio in the diagnosis of this entity.

NICTH is commonly present in the fifth to sixth decade of life with mean duration of symptoms ranging from weeks to months (3) before diagnosis. In our series, the mean age was 52.2 years, with a mean duration of symptoms for 24 weeks (2–78 weeks). Hypoglycemia can be either a presenting symptom (50%) of a tumor or may present later during the course of the disease (50%). In three of our patients, symptoms of hypoglycemia led to the discovery of the tumor. In the other two cases, the tumor was already known and symptoms of hypoglycemia were misinterpreted as psychiatric illness during the period of observation and treatment. Histologically identical tumor resected in the past without any evidence of hypoglycemia has been reported in few of the NICTH patients (3). We observed this in one of our patients (case 1). He had presented with a similar tumor 10 years earlier without hypoglycemia, which was resected and presented again with fibroma causing hypoglycemia, but was located at a different site. In patients with NICTH, the symptoms of hypoglycemia usually occur between meals or in the morning. The clinical presentation could be bizarre, as seen in two of our cases. Therefore, in any patient with large mesenchymal or epithelial tumor with vague neuropsychiatric manifestations without vascular events or brain metastasis, NICTH should always be considered. In addition to hypoglycemic symptoms, acromegaloid facial features, extreme oiliness of the skin, and rhinophyma have also been described (4). These features were present in two of our patients and are usually reversible following treatment of underlying disease. In IGF2 acting through IGF1 receptor, a phenomenon of specificity ‘spill over’ may contribute to the development of acromegaloid features (5). The other common presenting complaints that may lead to detection of these tumors are weight loss, abdominal mass, and pain.

In NICTH patients, the serum levels of insulin, C-peptide, and IGF1 are usually decreased or undetectable; however, the circulating level of total IGF2 as determined by conventional immunometric or receptor assays may be increased, decreased, or normal. These conventional assays can only determine the circulating levels of total IGF2. Total IGF2 reflects combination of variants of IGF2-like ‘big IGF2’ or immature IGF2, which is a 10–17 kDa fraction and mature IGF2 which is a 7.5 kDa form. These variants of IGF2 can only be detected by BIOGEL P-60 column chromatography. ‘Big IGF2’ normally contributes only 10–20% to the total pool of IGF2, but in patients of NICTH, the contribution increases to 60–70% of the total IGF2. This chromatography-based detection method, although gold standard, is usually expensive, time consuming, and is not easily available. So, in patients with hypoinsulinemic hypoglycemia, low IGF1 and a normal total IGF2 by conventional method, the ratio of IGF2:IGF1 of >10 is a useful additional marker and it was helpful in four of our five patients. Serum IGF1 levels were measured using ELISA method in all our patients. Another pointer toward the diagnosis of NICTH is low GH. This is especially useful in resource-poor settings where the facility for IGF2 is not easily available. ‘Big IGF2’ competes with IGF1 for binding to IGF1 binding proteins (IGFBPs), resulting in an increase in the free fraction of IGF1. This increased fraction of free IGF1 feedbacks negatively resulting in low GH (6). Hypokalemia has been described in 53% of patients in a previous series from Japan (7). This finding was observed in one of our patients. This is due to insulin-like activity of IGF2. The tumors causing NICTH are usually very large. They are well differentiated, slow growing, benign, or malignant; about 5–10 cm in size; and weigh an average of 2–4 kg at the time of surgery. They are easily detected by conventional radiological imaging. In our study, the mean tumor size was 16.4 cm and mean tumor weight was 3.6 kg. Functional imaging like FDG–PET may lead to a false negative result due to accelerated uptake of FDG by skeletal and cardiac muscles as these tissues have a much higher expression of insulin receptor when compared with the tumor tissue. Therefore, FDG–PET is a poor method to evaluate relapse of the disease and metastasis in these patients. Of the wide range of tumors present with NICTH, 40% could be tumors of mesenchymal origin like mesothelioma, hemangiopericytoma, solitary fibrous tumor, leiomyosarcoma, gastrointestinal stromal tumor, and fibrosarcoma. Another 40% could be due to tumors of epithelial origin, e.g. hepatocellular carcinoma, carcinoma of the stomach, lung, colon, prostate, neuroendocrine, and hematopoietic in origin. For the remainder, the origin is unknown (3). In our series, four patients had mesenchymal tumor and one had epithelial tumor. Therapeutic options for NICTH include complete removal of the tumor or debulking followed by radiotherapy and chemotherapy for inoperable disease. Glucocorticoid therapy is the most effective medical treatment for long-term relief (8) (9) (10). It ameliorates hypoglycemia by increasing gluconeogenesis and by suppressing the production of ‘big’ IGF2. Recombinant human GH has also been tried and it stimulates hepatic gluconeogenesis, increases serum IGFBP3 and ALS, thereby decreasing free IGF2 to act on insulin receptors. However, it has only modest efficacy and the dose required is very high (9). The utility of GH may be compromised in patients having concurrent pseudoacromegalic features.

In conclusion, NICTH should be considered in patients who present with recurrent hypoglycemia associated with suppressed serum insulin and IGF1 levels. In the absence of IGF2 assays, low serum insulin in combination with low IGF1 levels at the time of hypoglycemia is helpful in making the diagnosis of NICTH. Complete removal of the tumor is the mainstay of treatment. Glucocorticoid therapy may be useful when tumor is inoperable or in the presence of residual disease.

## Patient consent

Written informed consent has been obtained from the surviving patients for publication of the submitted case report and accompanying images.

## Author contribution statement

Dr P Dutta clinically managed all five patients, drafted the manuscript, and also edited the manuscript. Dr A Aggarwal helped in the management of case 5 and editing of the manuscript. Drs Y Gogate and V N Shah helped in the management of cases 3 and 4. Dr U Nahar reported on the histopathological findings of all the five patients. Dr M Singla helped in the management of case 5. Dr N Khandelwal reviewed the anatomical scans (CECT) of all patients and he also did the reporting. Dr A Bhansali helped in the management of all the five patients and he did the final editing of the manuscript.
